# The dual effect of pharmacological ascorbate on radiation: The best of both worlds

**DOI:** 10.18632/oncotarget.26396

**Published:** 2018-11-30

**Authors:** Joseph J. Cullen

**Affiliations:** Professor of Surgery, Departments of Surgery and Radiation Oncology, The University of Iowa Holden Comprehensive Cancer Center, and The Veterans Affairs Medical Center, Iowa City, IA, USA

**Keywords:** pancreatic cancer, pharmacological ascorbate, radiation therapy, radioprotection, radiosensitization

Pancreatic cancer is the fourth leading cause of cancer death in the United States and is increasing in incidence with a dismal prognosis [1]. Treatments include surgical resection for curative intent, which may occur in less than 3% [2]; chemotherapy with a response of 30% [3]; and an overall survival of 11-13 months with radiation therapy for locally advanced disease [4]. Because of the lack of therapeutic responsiveness of pancreatic cancer to surgery, chemotherapy, and radiation therapy, survival beyond five years is rare with median survival less than six months. Thus, novel and effective therapies directed against pancreatic cancer are needed to control progression and metastatic disease.

Ascorbate (vitamin C) is one of the early unorthodox therapies for cancer, based on unsupported hypotheses promoted by Cameron and Pauling. A prospective study was conducted that randomized patients to ascorbate treatment or palliative therapy with treated patients having a median survival of 343 days *vs.* 180 days for controls [5]. To test whether ascorbate was effective, two randomized placebo-controlled studies randomized to oral ascorbate were conducted but neither study showed benefit [6, 7]. Subsequently, ascorbate treatment was considered useless. However, it was not recognized that oral and intravenous ascorbate has strikingly different pharmacokinetics. Cameron gave patients ascorbate intravenously as well as orally, while Moertel's patients received only oral ascorbate. Infusing ascorbate (pharmacological ascorbate, high dose ascorbate) at 50-100 grams intravenously achieves plasma levels of 15-25 mM [8], which is selectively cytotoxic to cancer cells *in vitro* and *in vivo* [9]. Pharmacological ascorbate-mediated cell death has been shown to be due to hydrogen peroxide (H2O2) generation, *via* ascorbate radical formation, with ascorbate as the electron donor [9]. More recently, the radio-sensitizing effects of pharmacological ascorbate have been clarified. Our group's recent study demonstrates great potential for the use of pharmacological ascorbate as a radiosensitizer in pancreatic cancer by enhancing pancreatic tumor cell radiation cytotoxicity, while offering potential protection from radiation damage in normal surrounding tissue, making it an optimal agent for potentially improving locally advanced pancreatic adenocarcinoma [10]. In addition, pharmacological ascorbate in a first-in-human phase 1 clinical trial combined with radiation and gemcitabine for locally advanced pancreatic cancer demonstrated that this combination is safe, tolerable, and potentially efficacious (Figure 1) [10].

**Figure 1:**
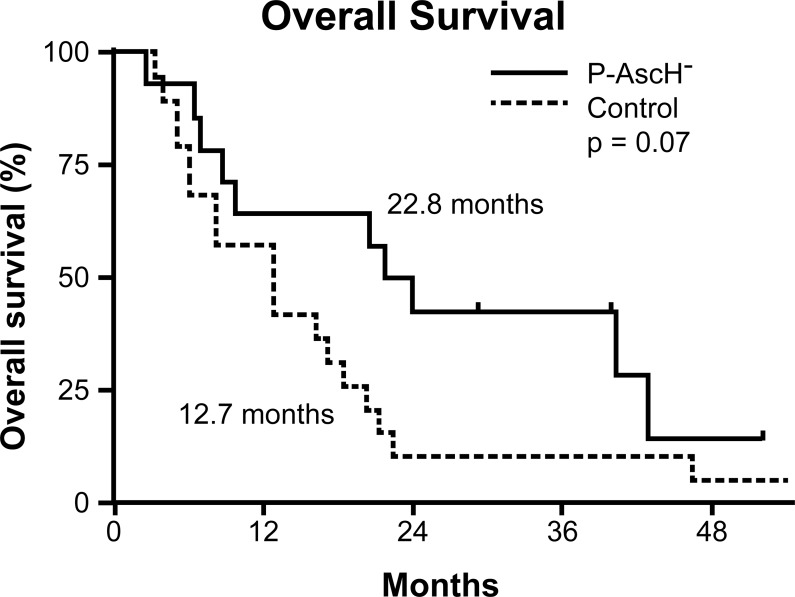
Survival as of December 2018 Median overall survival (OS) of subjects treated with pharmacological ascorbate, gemcitabine, and radiation was 22.8 months compared to a median OS of 12.7 months in the comparator arm of the study (*p* = 0.07). This rate is also increased compared to historical median OS of 11.1 months.

What do we have to lose? Pharmacological ascorbate is safe with little side effects and can be given as an intravenous infusion as other chemotherapeutic treatments. A selective radiosensitizing (pro-oxidant) drug with a protecting effect (antioxidant) on surrounding tissue is novel and could potentially change current radiation dose limitations used, allowing more aggressive treatment of locally advanced pancreatic cancer. Clearly, phase II trials are needed and should be supported.

## References

[1] Siegel RL (2018). CA Cancer J Clin.

[2] Bramhall SR (1995). Br J Surg.

[3] Conroy (2011). N Engl J Med.

[4] Zhong J (2017). Cancer.

[5] Cameron E (1991). Med Hypotheses.

[6] Creagan ET (1979). N Engl J Med.

[7] Moertel CG (1985). N Engl J Med.

[8] Welsh JL (2013). Cancer Chemotherapy and Pharmacology.

[9] Du J (2010). Clinical Cancer Research.

[10] Alexander MS (2018). Cancer Research.

